# Woorara in Epilepsy

**Published:** 1861-06

**Authors:** 


*Woorara in Epilepsy.*—The Baltimore Journal of Medicine states, that M. Thiercelin presented a paper to the French Academy on the 12th of November last, giving his experience with Woorara, in the treatment of epilepsy. He reports two cases of several years' duration, which were greatly benefitted by the use of this agent.

